# Eine Malignität vorspiegelnde kombinierte gastrale und respiratorische Heterotopie im Rektum

**DOI:** 10.1007/s00292-025-01473-3

**Published:** 2025-10-09

**Authors:** Nina Siegl, Rupert Langer, Alexander Ziachehabi, Petar Noack

**Affiliations:** 1 Institut für Pathologie und Molekularpathologie, Kepler Universitätsklinikum und Johannes Kepler Universität Linz, Linz, Österreich; 2 Abteilung Interne IV – Gastroenterologie, Hepatologie und Endokrinologie, Ordensklinikum Linz, Linz, Österreich

**Keywords:** Endoskopie, Submukosale Dissektion, Gastrointestinaltrakt, Ektopie, Differentialdiagnose, Endoscopy, Endoscopic submucosal dissection, Gastrointestinal tract, Ectopia, Differential diagnosis

## Abstract

Heterotopie bezeichnet das Vorhandensein normaler Zellen oder Gewebe an anatomisch ungewöhnlichen Körperstellen. Im Gastrointestinaltrakt verursachen die meisten Fälle keine Symptome, können aber selten makroskopisch bzw. bei endoskopischen Untersuchungen mit Neoplasien verwechselt werden. Ein 45-jähriger Mann stellte sich mit Unterleibsschmerzen und Blut im Stuhl vor. Makroskopisch zeigte sich eine polypoide Läsion, die mittels endoskopischer Submukosadissektion in toto entfernt wurde. Histologisch zeigten sich ektope Magenschleimhaut, speicheldrüsenartige Strukturen vereinbar mit Bronchialdrüsen und respiratorisches Epithel. Diese Kombination ektopischen Gewebes ist in der Literatur bisher nur sehr selten dokumentiert.

## Abstract (en)

Heterotopia refers to the presence of normal cells or tissue located in anatomically unusual sites of the body. In the gastrointestinal tract, most heterotopic lesions do not cause any symptoms, but rarely, they can be mistaken for neoplasms during endoscopic examinations. A 45-year-old man presented with lower abdominal pain and blood in the stool. Macroscopically, a polypoid lesion was found and resected by endoscopic submucosal dissection. Histologically, ectopic gastric mucosa, salivary gland-like structures compatible with bronchial glands, and respiratory epithelium were found. This combination of a gastric and respiratory ectopia has so far been rarely documented in the literature.

## Anamnese

Ein 45-jähriger Mann berichtete von Schmerzen im linken Unterbauch sowie einer kurzzeitigen Episode mit Blut im Stuhl. Familienanamnestisch ist bei der Mutter eine Divertikulitis sowie bei der Großmutter ein kolorektales Karzinom bekannt. Der Patient hatte keine Vorerkrankungen und nahm auch keine Medikamente ein.

## Untersuchung

**Abb. 1:**
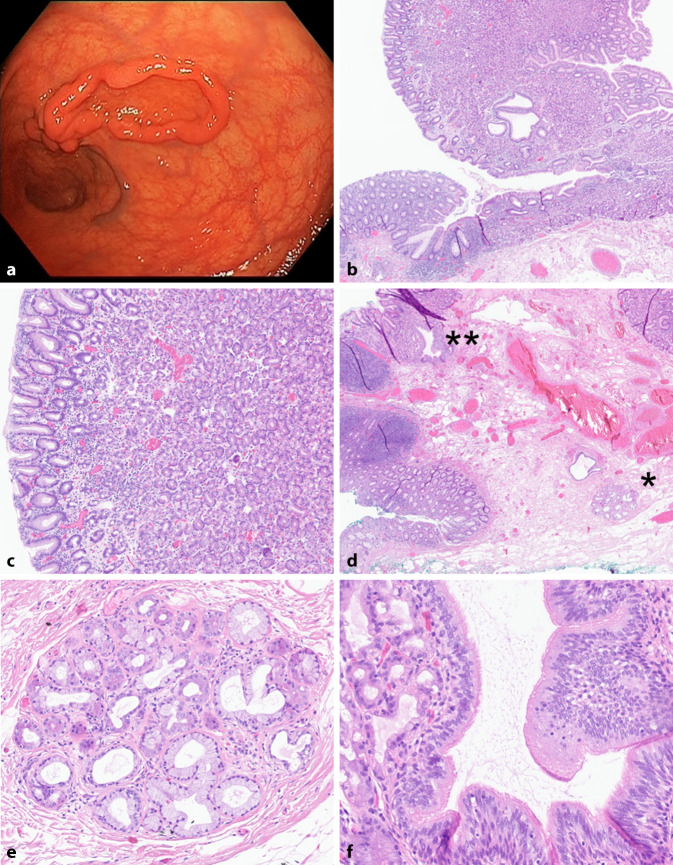
**a** Endoskopieaufnahme der polypoiden Läsion. **b** Dickdarmschleimhaut *links*, polypartige Schleimhautvorwölbung mit Magenschleimhaut vom Corpustyp *rechts.* Vergr. 20:1. **c** Magenschleimhaut vom Korpustyp. Vergr. 40:1. **d** Dickdarmschleimhaut, respiratorisches Epithel *links oben* (*Doppelstern*) sowie in Bindegewebe eingelagerte seromuköse speicheldrüsenartige Strukturen, vereinbar mit Bronchialdrüsen, *rechts unten* (*Stern*). Vergr. 20:1. **e** Seromuköse speicheldrüsenartige Struktur, vereinbar mit Bronchialdrüse – siehe *Stern* in **d**. Vergr. 100:1. **f** Respiratorisches Epithel mit Zylinderzellen und Kinozilien – siehe *Doppelstern* in **d**. Vergr. 200:1

Aufgrund von Schmerzen im linken Unterbauch und Blut im Stuhl fand im niedergelassenen Bereich eine Koloskopie statt, bei welcher ein max. 5 cm großer, schüsselartiger Polyp im Rektum festgestellt und der Patient daraufhin ohne weitere bioptische Abklärung ins Klinikum überwiesen wurde. Im Rahmen der stationär durchgeführten Koloskopie zeigte sich die Läsion im distalen Rektumdrittel, 4 cm von der Linea dentata entfernt, als flach-breitbasig und hyperplastisch imponierender Polyp mit wallartigem Rand (Abb. 1a). Der Polyp wurde in Narkose durch endoskopische Submukosadissektion (ESD) reseziert.

## Diagnostik

Im histologisch komplett aufgearbeiteten Material fanden sich mikroskopisch neben lokotypischem Gewebe an der Stelle der polypopoid imponierenden Struktur Magenschleimhautanteile vom Korpustyp mit mäßig hyperplastischen Foveolen, darüber hinaus seromuköse speicheldrüsenartige Strukturen, die Bronchialdrüsen zuordenbar sind, und von respiratorischem, zilientragendem Epithel ausgekleidete Gangstrukturen. Ferner zeigten sich abschnittsweise geringe bis mäßige chronische Entzündungsinfiltrate. Es gab keinen Hinweis auf Dysplasie oder auf einen malignen invasiven Prozess. Die ektope Magenschleimhaut und die seromukösen Speicheldrüsenanteile waren jeweils randbildend im Bereich der Präparatenden. Die Diagnose einer kombinierten gastralen und respiratorischen Ektopie im Rektum wurde gestellt.

## Therapie und Verlauf

Am Folgetag zeigten sich in der Laborkontrolle mit Ausnahme einer Hypercholesterinämie keine Auffälligkeiten, wodurch der Patient in gutem Allgemeinzustand aus dem Krankenhaus entlassen werden konnte. In einer 8 Monate nach der ESD durchgeführten Rektoskopie zeigte sich eine blande Narbe der endoskopischen Submukosadissektion. Der digital-rektale Tastbefund war unauffällig und der Patient war seit der Resektion der Läsion beschwerdefrei. Weitere Koloskopien werden laut Vorsorgeleitlinien empfohlen.

## Diskussion

Heterotopien sind eine seltene, aber wichtige Differentialdiagnose bei Läsionen im Gastrointestinaltrakt, die insbesondere in endoskopischen Untersuchungen mit (malignen) Neoplasien verwechselt werden können. Weshalb es zu Heterotopien kommt, ist noch nicht vollständig geklärt. Es gibt jedoch einige Theorien, wie ektopes Gewebe im Gastrointestinaltrakt entsteht. Möglicherweise liegen den Heterotopien entwicklungsbedingte Fehler zugrunde, es könnten aber auch fehlerhafte Regenerationsprozesse nach Schädigung der Mukosa eine Rolle spielen. Die Theorie der totipotenten Zellen bildet den dritten Erklärungsversuch [1].

Die häufigste Form der Heterotopie im Gastrointestinaltrakt ist die gastrale Heterotopie. Wesentlich seltener findet sich eine respiratorische Heterotopie mit respiratorischem Flimmerepithel wie im vorliegenden Fall [2, 3]. Eine Kombination aus gastraler und respiratorischer Ektopie, zu der hier auch die seromukösen Speicheldrüsenanteile gehören dürften, darf als Seltenheit gelten. Es wird diskutiert, dass diese Veränderung möglicherweise heterotopem embryonalem Vorderdarm(„foregut“)-Gewebe entspricht [4]. Ektopes Gewebe verursacht in der Regel keine Symptome. Nur in wenigen Fällen kommt es zu Komplikationen, wie z. B. Entzündungen, Blutungen, Perforationen oder Ulzerationen, sehr vereinzelt können aus Heterotopien auch Tumoren hervorgehen [4]. In der Regel stellen Heterotopien Zufallsbefunde dar und bedürfen keiner weiteren Therapie. Lediglich in Fällen mit klinischer Symptomatik sind (chirurgische) Interventionen indiziert, Rezidive sind selten, eine entsprechende Nachsorge jedoch empfohlen [5].

## Fazit für die Praxis

Im Rektum sind Heterotopien eine seltene und wichtige Differentialdiagnose bei polypoiden Läsionen und können v. a. endoskopisch mit (malignen) Neoplasien verwechselt werden. Der histologische Nachweis von organoid aufgebautem und weitgehend regelrechtem heterotopen Gewebe, wie z. B. gastraler Schleimhaut oder in seltenen Fällen auch respiratorischer Schleimhaut, stützt die Diagnose.
